# Eryptosis in Peritoneal Dialysis-Related Peritonitis: The Potential Role of Inflammation in Mediating the Increase in Eryptosis in PD

**DOI:** 10.3390/jcm11236918

**Published:** 2022-11-23

**Authors:** Grazia Maria Virzì, Sabrina Milan Manani, Davide Marturano, Anna Clementi, Silvia Lerco, Ilaria Tantillo, Anna Giuliani, Giovanni Giorgio Battaglia, Claudio Ronco, Monica Zanella

**Affiliations:** 1 Department of Nephrology, Dialysis and Transplant, St Bortolo Hospital, 36100 Vicenza, Italy; 2 International Renal Research Institute, Vicenza (IRRIV), 36100 Vicenza, Italy; 3 Department of Medicine (DIMED), University of Padua, 35100 Padua, Italy; 4 Department of Nephrology and Dialysis, Santa Marta and Santa Venera Hospital, 95024 Catania, Italy

**Keywords:** eryptosis, peritoneal dialysis, peritonitis, cytokines, inflammation

## Abstract

Background: Peritonitis and exit site infections are the main complications of patients treated with peritoneal dialysis (PD). Erythrocytes (red blood cells—RBCs) are very sensitive cells, and they are characterized by eryptosis (programmed cell death). The purpose of this research was to assess eryptosis in PD patients with PD-related peritonitis and its connection to inflammatory markers in vivo and in vitro. Material and Methods: In this study, we included 65 PD patients: 34 PD patients without systemic inflammation nor PD-related peritonitis in the previous 3 months, and 31 PD patients with an acute episode of PD-related peritonitis. We measured C-reactive protein (CRP) and cytokine (IL-1β, IL-6, and IL-18) levels as systemic inflammatory markers. Eryptosis was evaluated by flow cytometric analyses in freshly isolated RBCs. The induction of eryptosis due to in vitro exposure to IL-1β, IL-6, and IL-18 was verified. Results: Eryptosis was significantly higher in PD patients with peritonitis (9.6%; IQR 4.2–16.7), compared to the those in the other group (2.7%; IQR 1.6–3.9) (*p* < 0.0001). Significant positive correlations were noticed between eryptosis and CRP, IL-1β, and IL-6. RBCs, incubated with greater concentrations of all cytokines in vitro, resulted in significantly higher occurrences of eryptosis in comparison with those incubated with lower concentration and with untreated cell (*p* < 0.05), and for those with extensive exposure (*p* < 0.05). Conclusion: In conclusion, we investigated a potential relationship between systemic eryptosis and the in vivo and in vitro inflammatory damage of the peritoneal membrane during peritonitis. Thus, the presented results revealed that upregulated inflammatory markers and immune system dysregulation could be the cause of high levels of systemic eryptosis during PD-related peritonitis.

## 1. Introduction

Peritonitis and exit site infections are the main problems associated with peritoneal dialysis (PD) patients, characterizing 25% of all hospital admissions for the PD population [1,2,3]. Serious and recurrent PD-related peritonitis provokes long-term complications which may hypothetically result in peritoneal membrane damage [4] and the need to switch to hemodialysis.

Erythrocytes-RBCs are very sensitive cells, exhibiting a highly specific and well-organized membrane composition and structure, which respond to xenobiotic and endogenous agents [5,6,7]. RBCs are characterized by a specific type of programmed cell death, comparable to apoptosis, defined as eryptosis [5,6,7]. Eryptosis is involved in many clinical conditions, such as anemia, diabetes, uremia, sepsis, fever, and dehydration [5,8,9,10,11,12,13]. Eryptosis is a biological process with intricate control, described by cell shrinkage, cell membrane blebbing, and scrambling with the exposure of the aminophospholipid phosphatidylserine (PS) on the outer membrane surface of the RBCs. PS exposed to the RBCs may adhere to the surface of the endothelial cells and macrophages, quickly engulfing the PS-marked RBCs. In this context, PS-exposed RBCs are degraded and cleared from circulation [12,14,15]. Specific signals inducing eryptosis include situations such as increased cytosolic Ca(2+) concentration, oxidative stress, inflammation, and the presence of uremic toxins [6]. Little is reported about eryptosis in PD patients, but recent articles have demonstrated higher eryptosis levels in PD patients in comparison with healthy subjects [16,17,18,19]. To the best of our knowledge, the physiological and pathological function of eryptosis in patients affected by PD-related peritonitis and its connection to the inflammatory pathways have not yet been explored in this population. The goal of this research was to assess the eryptosis levels in PD-related peritonitis and their association with systemic inflammatory markers in in vivo and in vitro settings. In particular, we expanded and confirmed our previous data [20] regarding eryptosis levels in healthy RBCs exposed to several concentrations of cytokines (IL-6, IL-1β, and IL-18) at different time points.

## 2. Materials and Methods

### 2.1. Patients

This cross-sectional study was carried out in the PD Center at San Bortolo Hospital in Vicenza, Italy. All subjects (>18 years old) regularly treated by PD for a minimum of 3 months were consequently eligible for this research. In total, 85 sequential PD patients were screened, and 20 of them were excluded due to an active systemic infection (other than peritonitis), such as autoimmune disease, malignancy, unstable angina, or end-stage cardiac, pulmonary, or hepatic disease.

**Figure 1:**
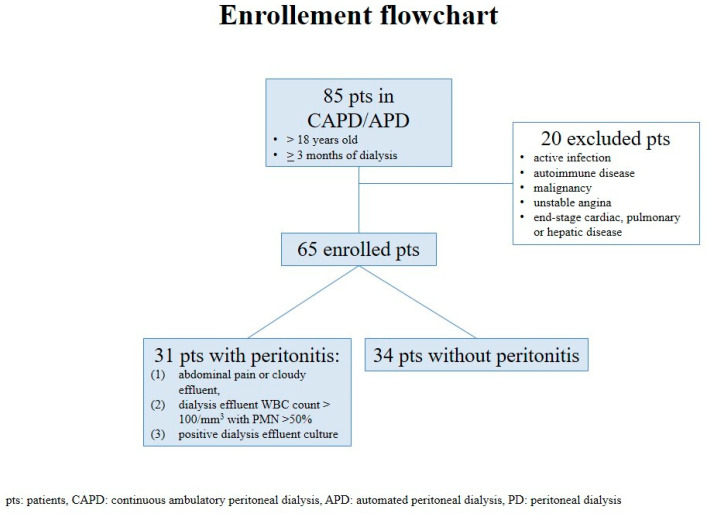
Enrollment flowchart of the study. WBC: white blood cell. PMN: Polymorphonuclear leukocytes.

We finally enrolled 65 PD patients and divided them into two groups: 31 PD patients presenting with acute PD-related peritonitis and a control group of 34 PD patients without any history of systemic inflammation nor peritonitis in the previous 3 months (Figure 1). Clinical, biochemical, and PD-related features were reported for all included subjects.

Peritoneal dialysis-related peritonitis was established according to the Guidelines of the International Society of Peritoneal Dialysis [1]. The peritoneal white blood cell (WBC) count was tested using the overnight collection of peritoneal effluent in all included subjects for 15 days. Relapsing peritonitis was defined as the development of peritonitis within 4 weeks of the completion of antibiotic treatment for a prior episode with the same organism isolated, or one sterile episode. Refractory peritonitis was defined as unsuccessful effluent clearing after 5 days of correct antibiotic use, according to current guidelines [1]. For relapsing and refractory peritonitis, samples were taken from the patients during the first day of peritonitis presentation.

### 2.2. Collection of Biological Specimens

Blood samples from the PD control group were drawn during the regular outpatient visits. On the contrary, blood samples were collected at the time of peritonitis diagnosis for patients presenting PD-related peritonitis. Biochemical parameters and C-reactive protein (CRP) levels were measured using ordinary laboratory techniques with an automatic analyzer (Dimension Vista, Siemens Healthcare, Tarrytown, NY, USA) in our Central Laboratory.

### 2.3. Cytokine Enzyme-Linked Immunosorbent Assay (ELISA)

The quantification of plasma cytokines (IL-1β and IL-6) was performed using the Human Instant ELISA kit (eBioscience, San Diego, CA, USA). Optical density was read at 450 nm, and the analysis was performed using a VICTORX4 Multilabel Plate Reader (PerkinElmer Life Sciences, Waltham, MA, USA). The concentration values for these molecules were extrapolated from standard curves. We repeated the tests in triplicate.

### 2.4. In Vitro Exposure to Cytokines and Induction of Eryptosis

We exposed healthy RBCs in vitro to cytokines and tested the induction of eryptosis. We dissolved the cytokine powders (Sigma, St. Louis, MO, USA) in complete cell medium [RPMI 1640 with stable L-glutamine (International PBI Italy, Milan, Italy) and 10% fetal bovine serum (Sigma)] to obtain a final concentration of 2000 ng/µL for IL-6 and IL-18, and 1000 ng/µL for IL-1β. Serial dilutions were constructed with the same medium (IL-6 and IL-18: 2000–1000–500–250–125–62.5–31.25–0 ng/µL and IL-1β: 1000–500–250–125–62.5–31.25–15.63–0 ng/µL). A total of 1.5 µL of RBCs were plated per well in 48-well plates in 300 µL of complete RPMI and exposed to incremental concentrations of IL-6, IL-18, and IL-1β for 4, 8, 12, and 24 h in RPMI 1640 under normal conditions (37 °C in 5% CO_2_). We adopted untreated RBCs as a negative control. Each cytokine dilution concentration was tested in duplicate.

### 2.5. Eryptosis Evaluation

All eryptosis determinations were carried out using freshly isolated RBCs. The cell volume was evaluated by flow cytometry and forward scattering (FS). PS avidly binds annexin-V, which is employed to detect eryptotic cells [15,20]. Thus, PS exposure at the RBC surface was estimated from FITC-AnnexinV binding (Beckman Coulter, Brea, CA, USA) by Navios Flow Cytometer (Beckman Coulter, Brea, CA, USA). RBCs were gated and counted by identifying those cells that exposed PS at the RBC surface. A total of 100.000 events were collected from each sample.

### 2.6. Statistical Analysis

Statistical analysis was performed using the SPSS software package. A *p*-value of <0.05 was defined as statistically significant. Categorical variables were reported as percentages; continuous variables were reported as mean ± standard deviation (parametric variables) or as the median and interquartile range (IQR) (nonparametric variables). The Mann–Whitney U test or T test was applied to compare the two groups, as appropriate. The Kruskal–Wallis test or the ANOVA test for multiple comparisons were used to compare the groups, when appropriate. Spearman’s rho correlations were tested to verify the correlation between variables. 

## 3. Results

### 3.1. Baseline Characteristics of the Subjects

A group of 65 chronic PD patients were included in this research. A total of 57% of the patients were treated with continuous ambulatory PD (CAPD) and 43% with automated PD (APD). The median PD vintage was 38 months (IQR 3.6–124.9 months). In our cohort, chronic kidney disease was related to diabetic nephropathy (15 patients), hypertension (16 patients), glomerulosclerosis (11 patients), nephroangiosclerosis (4 patients), polycystic kidney disease (4 patients), vesicoureteral reflux (3 patients), hemolytic uremic syndrome (2 patients), or other causes (10 patients). The clinical, biochemical, and PD-related features of the 65 enrolled subjects are listed in Table 1 (Table 1).

We split the PD patients into two groups: 34 PD patients without any history of systemic inflammation and/or peritonitis in the previous 3 months (control group, 27 males and 7 females, mean age 64 ± 14 years), and 31 PD patients with an acute episode of PD-related peritonitis, without any history of other systemic inflammation (19 male and 12 females, mean age 61 ± 15 years). At the time of PD-related peritonitis diagnosis, we evaluated the WBC count in a dialysate effluent (median WBC count 3578 × 106/L, IQR 1601–8159 × 106/L). 

### 3.2. No Patients Received Kidney Transplantation in This Period of Time

Table 2 reports the clinical, PD-related, and laboratory (conventional and unconventional) parameters of the two selected PD groups (Table 2).

### 3.3. Responsiveness in Patients with an Acute Episode of Peritonitis

All 31 PD patients affected by peritonitis were promptly treated with intraperitoneal broad-spectrum antibiotics, according to current guidelines. PD patients clinically recovered from peritonitis after 13.5 ± 5.4 days, on average. A total of 25/31 (80.6%) of the subjects experienced a first episode of peritonitis and responded to first-line antibiotics, whereas 6/31 (19.4%) had a relapsing episode of peritonitis, but subsequently responded to a second course of intraperitoneal antibiotics. Six subjects required catheter removal because of refractory peritonitis and were transferred to hemodialysis. 

### 3.4. Cytokines Evaluation

CRP and cytokine values were quantified in the plasma of both PD groups at the specific recruiting time. Inflammatory factors and cytokines concentrations are reported in Table 2 (Table 2). The median levels of these substances were significantly increased in subjects with PD-related peritonitis in comparison to PD subjects without peritonitis (*p* < 0.0001). 

We observed a significant positive correlation between CRP and IL-1β (Spearman’s rho = 0.36, *p* = 0.004) and IL-6 (Spearman’s rho = 0.25, *p* = 0.05). Additionally, IL-6 levels presented a positive correlation with IL-1β (Spearman’s rank = 0.49, *p* < 0.001). 

### 3.5. In Vitro Stimulation of Eryptosis Resulting from Cytokines Exposure

**Figure 2:**
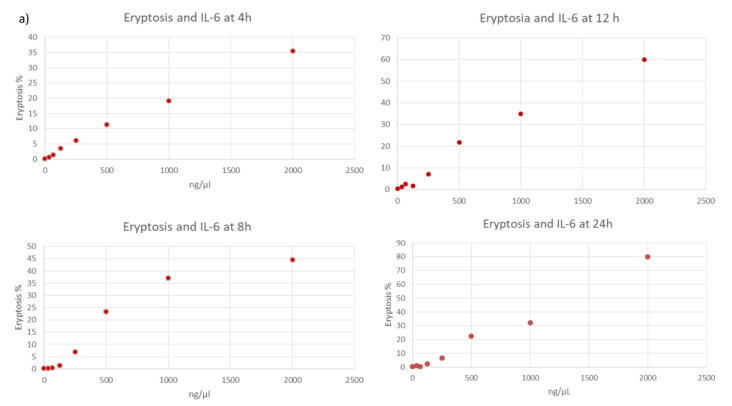

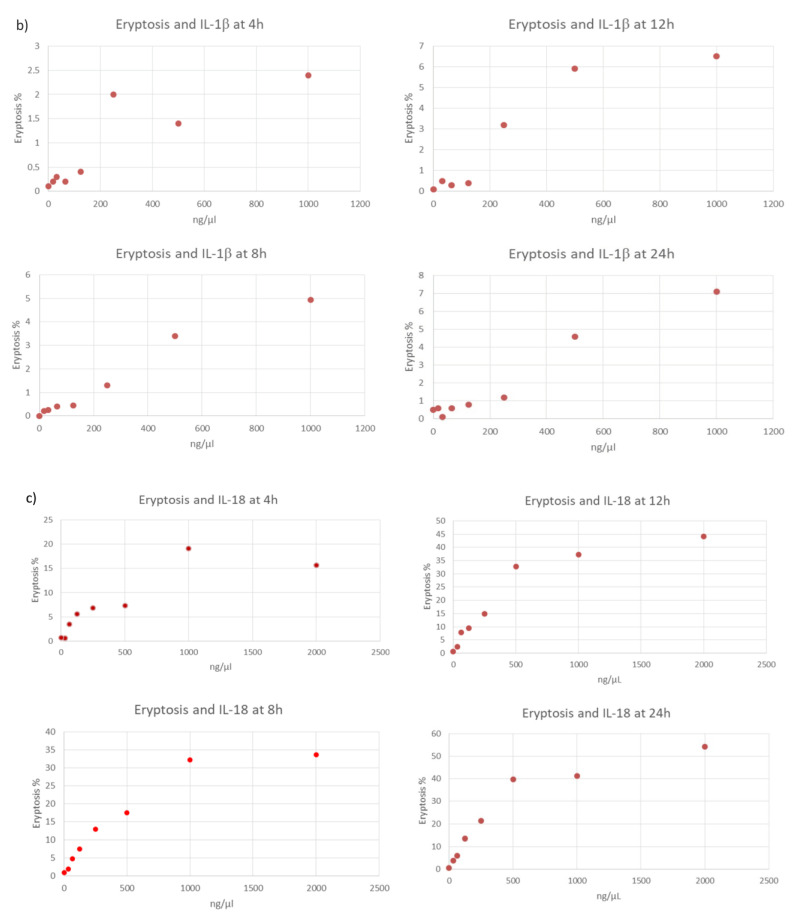
In vitro trigger of eryptosis by cytokines (**a**) IL-6, (**b**) IL-1β, and (**c**) IL-18 at 4, 8, 12, and 24 h.

The cytotoxic influence of cytokines was studied in vitro on healthy RBCs at different time points (4, 8, 12, and 24 h). RBCs incubated with IL-6, IL-18, and IL-1β demonstrated a significant increase in eryptosis. Cytofluorimetric results show significantly increased rates of eryptosis in healthy RBCs incubated with greater concentrations of cytokines in comparison with other lower concentrations and negative control/untreated cells/healthy RBCs (*p* < 0.05). Figure 2 describes eryptosis inducted by the in vitro exposure to cytokines at several dilutions of these selected molecules and at different time points (Figure 2).

### 3.6. RBCs Evaluation

**Figure 3:**
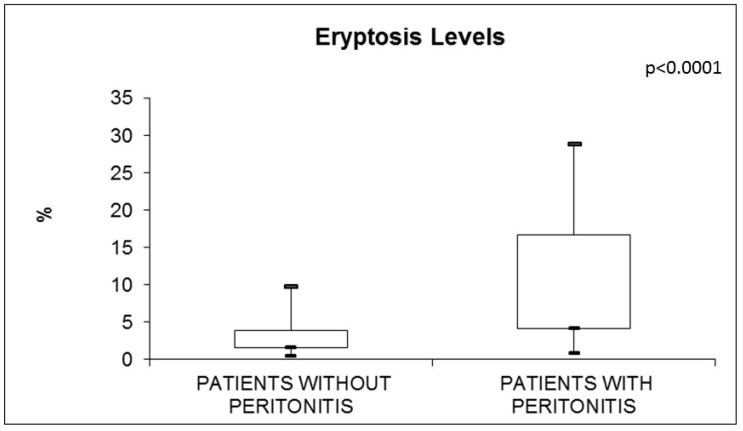
Eryptosis in PD subjects, with and without peritonitis.

Cell shrinkage, cell membrane scrambling, and PS exposure at the RBC surface are typical signals of the eryptotic process. Eryptototic RBCs were identified and quantified by cell volume dimension and AnnexinV-binding through flow cytometric assay. Generally, the RBCs of all PD subjects were dramatically deranged in their morphology. In particular, RBCs from PD patients with peritonitis were characterized by dramatically deranged morphology and by increased median cell volume (based on FS evaluation) in comparison with another PD groups (*p* < 0.001). The percentages of AnnexinV-binding RBCs were significantly increased in the peritonitis group (9.6%; IQR 4.2–16.7 vs. 2.7%; IQR 1.6–3.9) (*p* < 0.0001) (Figure 3). In Table 3, we report significant positive correlations regarding eryptosis levels and conventional and unconventional inflammatory indices, such as CRP, IL-6, and IL-1β (Table 3).

**Figure 4:**
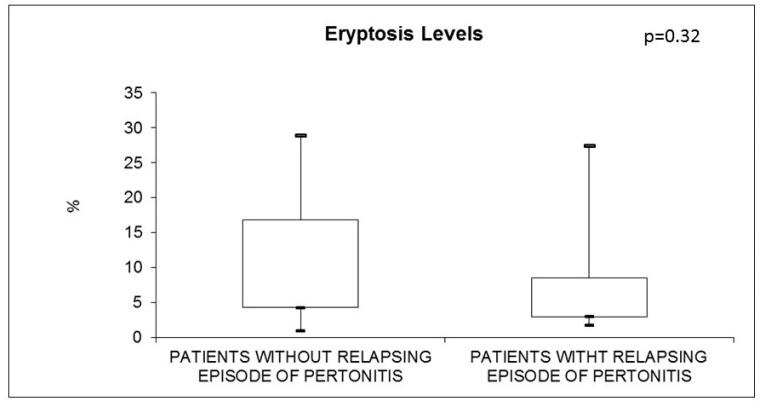
Eryptosis in PD subjects with (n = 6) and without (n = 6) relapsing episodes of peritonitis.

**Figure 5:**
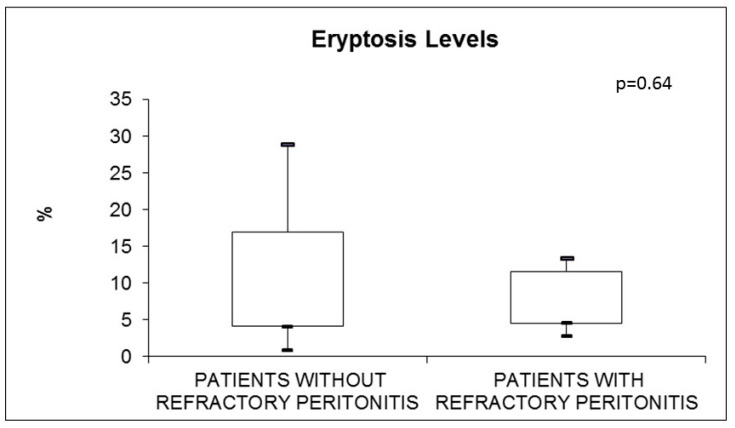
Eryptosis in PD subjects with (n = 6) and without (n = 25) refractory peritonitis.

Additionally, we analyzed subjects with PD-related- peritonitis. There was no significant difference in the value of eryptosis in patients with or without relapsing episodes of peritonitis (*p* = 0.32) (Figure 4). In the same way, eryptosis did not differ in subjects with or without refractory episodes of peritonitis (*p* = 0.64) (Figure 5).

## 4. Discussion

To our knowledge, this is the first demonstration of alterations in eryptosis during peritonitis in the PD population in comparison with PD-stable patients. Furthermore, this study reported and confirmed the association between eryptosis and inflammation during peritonitis. In particular, in this research, we investigated the eryptosis levels in PD-related peritonitis and we evaluated the relationship between eryptosis and conventional and unconventional inflammatory indices in PD patients with peritonitis in in vivo and in vitro settings (confirmation and integration of previous results) [20].

The central concern during PD is the protection of the peritoneal membrane. The activation of the inflammatory pathways and the presence of cytokines and oxidative stress molecules provoke histological and functional modifications of the peritoneal membrane. These alterations may affect peritoneal ultrafiltration failure and contribute to mortality risk [21]. Moreover, a protracted systemic inflammation after apparent clinical remission of peritonitis was reported. Specifically, Lam et al. observed that PD patients have a persistent elevation of CRP, which is evocative of chronic inflammation, even 6 weeks after the apparent clinical remission of the PD-related peritonitis [21]. 

In this research, we included 31 PD patients with the diagnosis of acute peritonitis and a control group composed of 34 PD patients without any history of systemic inflammation or peritonitis in the previous 3 months. We evaluated eryptosis percentage, CRP, IL-6, and IL-1β levels in the PD controls and in the PD patients with peritonitis on the first day of peritonitis. Our data demonstrated enhanced levels of inflammatory indices and increased eryptosis in the PD patients with peritonitis in comparison with the PD controls. In particular, the percentage of eryptosis was 3 times higher in PD patients with peritonitis. In addition, we noticed a positive correlation between all inflammatory indices and eryptosis levels. 

Numerous studies support the role of immune system dysregulation and inflammatory pathway activation during eryptosis [9,22,23,24]. In our in vitro study, we visibly highlighted the cytotoxic effect of cytokines on healthy RBCs, showing that they induce elevated eryptosis. This in vitro-induced eryptosis was noticed for all time points and all concentrations, in comparison with untreated RBCs. Obviously, a higher eryptosis level was observed for RBCs treated with a higher concentration of IL-6, IL-18, and IL-1β. These results expanded (more cytokines and more time points) and confirmed data obtained from our previous studies regarding eryptosis induced by inflammation markers and uremic toxins [20]. In particular, our results are supported by Bester et al., who examined the role of IL-1β, IL-6, and IL-8 upon the structure of erythrocytes and platelets [25]. All three interleukins were found to be responsible for the increased hypercoagulability of whole blood. Importantly, the RBC structure was particularly modified by the presence of IL-8, which causes evident structural modifications to the erythrocyte membranes and induces eryptosis initiation [25]. There is no evidence regarding the role of IL-8 in the trigger of eryptosis; nevertheless, it is a recognized stimulator of apoptosis, and it is closely involved in the traditional apoptotic pathways [25].

As a conventional marker of inflammation, we investigated CRP levels and their relationship with eryptosis. CRP has been reported to induce apoptotic mechanisms in nucleated cells [26]. Recently, Abed et al. investigated the effect of CRP on the eryptotic process [27]. They reported that CRP provokes PS translocation in RBCs; furthermore, treatment of healthy RBCs with different CRP concentrations, extrapolated from CRP levels present in subjects with acute appendicitis, causes PS translocation [27]. On the basis of our results, we confirmed CRP eryptosis induction and the strong relationship between CRP and eryptosis in conditions of acute inflammation, such as peritonitis and acute appendicitis.

In addition, we specifically investigated PD patients with peritonitis, but eryptosis levels did not differ between patients with relapsing and refractory peritonitis. We hypothesized that peritonitis itself is connected with eryptosis, and relapsing episodes or refractory peritonitis do not influence or alter eryptosis mechanisms.

It is known that during peritonitis, resident and infiltrating cells produce and release inflammatory molecules in the peritoneal cavity [4,28]. Moreover, recent reports demonstrated that inflammatory markers are also present in PD patients after the resolution of the peritonitis [4,21,29]. Furthermore, Brown et al. reported higher spike in total inflammatory cells and an increase in neutrophil counts (400-fold) in the peritoneal dialysis effluent up to 3 weeks after the clinical resolution of peritonitis [4]. Dialysate levels of cytokines, such as IL-1β, IL-6, and transforming growth factor-β (TGF-β), increase markedly on the first day of peritonitis, and then their concentrations decrease gradually. Inflammatory pathways remain active, with the production and release of inflammatory molecules, for at least 6 weeks from the time of clinical remission of peritonitis. In this situation, the peritoneal cytokine networks may theoretically affect the characteristics and function of the peritoneal membrane [21,29,30].

In this context, we investigated a possible link between systemic eryptosis and inflammation during peritonitis in vivo and in vitro. In particular, we theorized that the eryptosis enhancement is directly connected with peritonitis, and based on the results, we hypothesized that the injury of the peritoneal membrane could be related to eryptosis. Specifically, the presented results revealed that upregulated inflammatory markers and immune system dysregulation can be the cause of high levels of systemic eryptosis during PD-related peritonitis. Based on these data, eryptosis determination could be a new supplementary test to assist clinicians in the diagnosis of PD-related peritonitis. Furthermore, these pilot data can be defined as hypothesis-generating, and inspire additional investigations regarding eryptosis in PD patients with peritonitis. Future studies could include the monitoring of eryptosis during peritonitis, with particular attention paid to relapsing and refractory episodes, including other biomarkers [31]. These data offer evidence for extended research that may continue to elucidate eryptosis in PD patients, with and without peritonitis.

## Figures and Tables

**Table 1:** Clinical, laboratory, and PD-related parameters of all 65 patients included in the study.

	PD Patients
Male/Female	46M/19F
Age, years	62.8 ± 14.3
CVD	31/65
Diabetes	21/65
Months of dialysis	31.3, IQR 17.8–44.6
CAPD/APD	37/28
Weekly creatinine clearance, l/week/1.73 m^2^	57.9, IQR 46.9–71.8
Weekly Kt/Vurea	1.8, IQR 1.5–2.1
Serum creatinine, mg/dL	8.1, IQR 6.1–11.6
Urea, mg/dL	132, IQR 103–142
Uric acid, mg/dL	5.2, IQR 4.6–6.2
Hemoglobin, g/dL	11.5, IQR 10.8–12.1
WBC, ×10^9^/L	7.0, IQR 5.6–8.2

CVD: cardiovascular disease. IQR: interquartile range. Continuous ambulatory PD (CAPD). Automated PD (APD). WBC: white blood cells.

**Table 2:** Clinical PD-related parameters and conventional and unconventional inflammatory markers in patients with and without peritonitis.

Clinical Features			
	Patients with Peritonitis (n = 31)	Patients without Peritonitis (n = 34)	*p*-Value
Male/Female	19/12 females	27/7 females	0.11
Age, years	61.0 ± 14.9	64.1 ± 13.8	0.46
CVD	16/31	12/34	0.19
Diabetes	11/31	10/34	0.6
**Peritoneal Dialysis characteristics**			
	**Patients with Peritonitis** **(n = 31)**	**Patients with Peritonitis** **(n = 31)**	***p*-Value**
Months of dialysis	33.6, IQR 19.1–43.5	31.3, IQR 17.8–45.7	0.79
CAPD/APD	16CAPD/14APD	20CAPD/14APD	0.75
Weekly Creatinine Clearance	60.9, IQR 49.4–84.1	55.2, IQR 46.4–62.8	0.15
Weekly Kt/Vurea	1.7, IQR 1.5–1.9	1.8, IQR 1.6–2.1	0.21
Relapsing peritonitis n = 6			
Refractory peritonitis n = 6			
**Laboratory Parameters**			
	**Patients with Peritonitis** **(n = 31)**	**Patients with Peritonitis** **(n = 31)**	***p*-Value**
CRP, mg/dL	4.4, IQR 1.0–12.6	0.4, IQR 0.3–0.9	*p* < 0.001
IL-1β, pg/mL	36.3, IQR 3.1–58.3	1.4, IQR 0.8–2.5	*p* < 0.001
IL-6, pg/mL	173.4, IQR 107.2–206.7	19.8, IQR 9.4–61.3	*p* < 0.001

CVD cardiovascular disease. IQR: interquartile range. Continuous ambulatory PD (CAPD). Automated PD (APD).

**Table 3:** Spearman’s rho correlations and *p*-values for eryptosis percentage and inflammatory marker levels.

	Spearman’s Rho Correlation	*p*-Value
Eryptosis/CRP	0.46	<0.001
Eryptosis/IL-1β	0.42	<0.001
Eryptosis/IL-6	0.50	0.001

## Data Availability

All data generated or analyzed during this study are included in this article. Further inquiries can be directed to the corresponding author.
